# Microbiota-gut-brain Axis: Novel Potential Pathways for Developing Antiepileptogenic Drugs

**DOI:** 10.2174/1570159X23666250414094040

**Published:** 2025-04-15

**Authors:** Huifeng Li, Huanling Lai, Yue Xing, Shangnan Zou, Xiaofeng Yang

**Affiliations:** 1 Department of Neurology, The First Affiliated Hospital of Guangzhou Medical University, 151 YanJiangXi Road, Guangzhou 510120, Guangdong Province, China;; 2 Guangzhou National Laboratory, No. 9 XingDaoHuanBei Road, Guangzhou International Bio Island, Guangzhou 510005, Guangdong Province, China;; 3 Department of Neurology, The Seventh Affiliated Hospital of Sun Yet-sen University, No. 628, Zhenyuan Road, Xinhu Street, Guangming District, Shenzhen 517108, Guangdong Province, China

**Keywords:** Gut microbiota, microbiota-gut-brain axis, epilepsy, antiepileptic drug, antiseizure medication, epileptogenesis, epilepsy treatment

## Abstract

The treatment of epilepsy remains imperfect due to a lack of understanding of its pathogenesis. Although antiseizure medications can control most seizures, up to 30% of patients experience uncontrolled seizures, leading to refractory epilepsy. Therefore, elucidating the pathogenesis of epilepsy and exploring new avenues to design antiepileptic drugs may improve epilepsy treatment. Recent studies have identified an imbalance of the gut microbiota (GM) in both patients with epilepsy and various animal models of epilepsy. In response to this phenomenon, an increasing number of studies have focused on controlling seizures by regulating GM homeostasis, utilizing methods such as dietary restrictions, fecal microbiota transplantation, and the use of prebiotics. Surprisingly, these interventions have shown promising antiepileptic effects, suggesting that GM, through the regulatory role of the microbiota-gut-brain axis (gut-brain axis), may emerge as a novel strategy for treating epilepsy. This review aims to discuss the research progress on the relationship between GM and epilepsy, incorporating the latest clinical studies and animal experiments. We will specifically concentrate on the potential key role of the gut-brain axis in epileptogenesis, epilepsy development, and outcomes of epilepsy. Through a detailed analysis of the underlying mechanisms of the gut-brain axis, we aim to provide a more comprehensive perspective on understanding the pathophysiology of epilepsy and lay the groundwork for the development of new antiepileptic drugs in the future.

## 1 INTRODUCTION

The etiologies of epilepsy are complex. Current research suggests that they include genetic and metabolic factors, infections, and structural lesions in the brain [1-5]. However, the cause of most epilepsy patients is unknown. Considering the importance of timely seizure control, epilepsy pharmacotherapy is an effective treatment strategy. Up to date, significant advances in epilepsy research and treatment have led to a significant increase in third-generation antiseizure medications (ASMs). Twenty-six drugs for epilepsy have been approved by the US Food and Drug Administration [6]. Despite this, seizures are not controlled in about one-third of epilepsy patients, leading to refractory epilepsy [7]. In addition, there are no ASMs that can prevent epilepsy or epilepsy modification. To develop effective treatments, an in-depth understanding of the pathogenesis of epilepsy is essential, and by relying on pathogenesis, we can help develop effective antiepileptic drugs (AEDs).

Recently, the microbiota-gut-brain axis (gut-brain axis) has become increasingly popular in neurological disorders due to its role in the bidirectional communication of neuronal, endocrine, metabolic, and immune pathways. Over the past 20 years, it has been discovered that the gut-brain axis affects cognitive, behavioral, and neuronal development [3, 8, 9]. Alterations in gut microbiota (GM) have been observed in many neuronal disorders, including depression, Parkinson’s disease (PD), Alzheimer's disease (AD) and autism [10-12]. Epilepsy is no exception. Studies comparing the GM of healthy and epileptic patients have consistently shown significant differences [13]. Furthermore, interventions targeting GM have demonstrated antiepileptic effects. Thus, the gut-brain axis holds great promise to expose the pathogenesis of epilepsy. In addition, remodeling or modulating GM may be an effective means of developing AEDs for epilepsy treatment. In this review, we summarize the relationship between GM and epileptogenesis and epilepsy development. Additionally, we analyze the potential of the gut-brain axis as a novel potential pathway for developing AEDs, providing new insights into the etiology and therapy of epilepsy.

## 2 CONCEPT OF MICROBIOTA-GUT-BRAIN AXIS

### 2.1 Gut Microbiota and Microbiota-gut-brain Axis

The term GM refers to the trillions of bacteria, viruses, fungi, and protozoa that colonize the gastrointestinal tract. The majority belong to the two phyla *Firmicutes* and *Bacteroidetes*, followed by *Proteobacteria*, *Actinobacteria*, *Fusobacteria, and Verrucomicrobia* [14]. The composition of GM is influenced by both genetic and environmental factors, including diet, geographic location, toxin exposure, and hormones [15]. Hundreds of species of bacteria in the human gut collectively encode about 100 times more genes than the human genome itself [16], suggesting that GM plays a critical role in human physiological functioning. Studies have shown that GM is mutually beneficial to the host due to its role in digestion, absorption, immunity, metabolism, and maintenance of the mucosal barrier in the gastrointestinal tract [17, 18]. Additionally, GM regulates the survival and promotes the regeneration of enteric neurons [19]. Thus, germ-free (GF) mice were reported to be more susceptible to inflammatory bowel diseases (IBDs) [20]. Furthermore, alterations in specific GM are important for the development of many diseases.

**Fig. (1):**
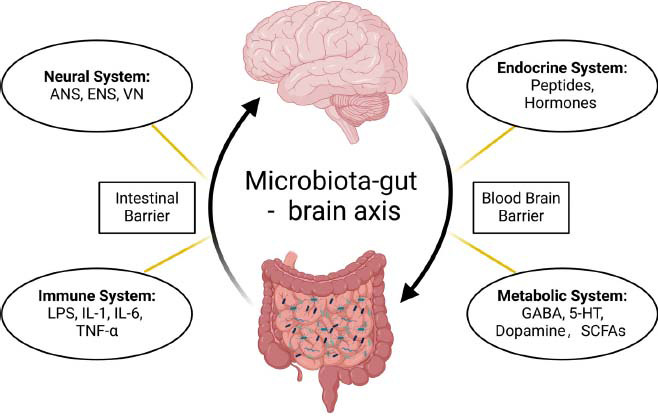
The Microbiota-gut-brain axis. Bidirectional communication between the gut and the brain includes neural, endocrine, immune, and metabolic systems through the intestinal barrier and the blood-brain barrier (BBB). Neural pathways include the autonomic nervous system (ANS), enteric nervous system (ENS), and vagus nerve (VN). Immune pathways include a range of inflammatory factors and cytokines, such as lipopolysaccharide (LPS), interleukins (IL)-1, IL-6, and tumor necrosis factor (TNF)-α. Endocrine and metabolic pathways encompass a variety of microbe-derived neuroactive compounds and metabolites, including peptides, gut hormones, gamma-aminobutyric acid (GABA), 5-hydroxytryptamine, and short-chain fatty acids (SCFAs). Created with BioRender.com.

The gut-brain axis refers to the bidirectional communication between the gut and the brain, including the nervous, endocrine, immune, and metabolic systems through the gut barrier and the blood-brain barrier (BBB), linking emotional and cognitive centers of the brain with peripheral intestinal functions [21-23] (Fig. **1**). It is crucial for maintaining homeostasis in both the gastrointestinal tract and the central nervous system (CNS) [24]. The brain regulates the gut by virtue of the autonomic nervous system (ANS) and hypothalamic-pituitary-adrenal (HPA) axis [25, 26]. The ANS consists of the sympathetic nervous system, parasympathetic nervous system, and enteric nervous system [27]. Notably, the vagus nerve is a major component of the parasympathetic nerve, which has abundant afferent and efferent nerves in the spinal cord, receiving and transmitting signals from the gut and the brain [28]. The HPA axis coordinates the stress response and produces signaling molecules that are distributed throughout the body, affecting GM [29]. Conversely, the gut can also signal to the CNS *via* the ANS, transmitters, and chemical metabolites [30]. The enteric nervous system is the most crucial component of ANS, also known as “the second brain,” contributing to gut-brain communication [31]. GM stimulates enteroendocrine cells to produce gut peptides and hormones, which are absorbed into the bloodstream and affect central events [32]. It can also produce neurotransmitters such as serotonin, dopamine, and gamma-aminobutyric acid (GABA) that signal the brain [32-34].

### 2.2 Effects of Gut Microbiota on the Nervous System Structure and Function

Research indicates that GM can exert significant effects on various processes within the nervous system, including brain myelination, neurogenesis, microglia activation, mood, and cognition [35-37]. Myelination, a developmental phenomenon that begins in the neonatal period and continues into early adulthood, aligns with the development of the gut-brain axis. Administration of antibiotics cocktail during the neonatal period has been found to result in long-lasting gut deficits, myelination dysregulation, and damage to oligodendrocyte and hippocampal neurogenesis, as well as behavioral deficits [38]. Alterations in the gut commensal microbiota have been linked to neuroinflammation [39]. It has also been reported that microglia are immune cells of the nervous tissue whose activation plays an important role in neurological disorders [40]. A study transplanting GM from mice subjected to prolonged unpredictable mild stress, characterized by an increased relative abundance of *Helicobacter*, *Bacteroides*, and *Desulfovibrio*, along with decreased relative abundance of *Lactobacillus*, *Bifidobacterium*, and *Akkermansia*, into naïve animals resulted in microglial priming in the recipient animals [41]. Furthermore, serotonin, a neurotransmitter primarily produced by enterochromaffin cells, is involved in the regulation of mood, behavior, and cognition [42], whose synthesis requires gut microbes to metabolize its precursor substance, tryptophan [43]. *Bifidobacterium dentium*, which colonizes the mucus layer of GF mice, was found to stimulate serotonin release from enterochromaffin cells through acetic acid release, consequently increasing serotonin and serotonin receptor levels in the brain [44]. Intestinal metabolites such as amino acids, SCFAs, bile acids, and hormones can modulate nervous system function by influencing the immune-inflammatory response and the integrity of BBB [45]. Additionally, GM dysbiosis leads to increased release of cytokines, such as interleukin (IL)-1, IL-6, and tumour necrosis factor (TNF)-α, as well as components of the bacterial cell wall like lipopolysaccharide (LPS) and peptidoglycan. These factors enhance BBB permeability and activate the HPA axis [46].

### 2.3 Gut Microbiota and Neurological Disorders

Dysbiosis of the GM affects the gut-brain axis and may lead to CNS disorders [47]. Over the past two decades, a large number of studies have emphasized the important role of GM in controlling the course of various neurological disorders (Table **1**). A study demonstrates that increased intestinal *Klebsiella pneumoniae* by gavage increases susceptibility to pentylenetetrazole (PTZ) -induced seizures in mice [48]. Another study analysed the composition of GM in 41 epilepsy patients and 30 family members of the patients. Epilepsy patients exhibited high levels of *Fusobacterium*, *Verrucomicrobia,* and *Alloprevotella* [49]. GF 3×Tg mice exhibited reduced pathological changes in amyloid beta plaques and neurofibrillary tangles in the brain, as well as improved cognitive function compared to SPF 3×Tg mice. Further findings demonstrated that a complex GM is necessary for behavioral deficits, microglia activation, and pathological changes in AD [50]. To determine whether intestinal dysfunction was responsible for the reduction in CNS GABA in essential tremor patients, fecal microbiota from both essential tremor patients and healthy individuals were transplanted into mice with essential tremor, respectively. Essential tremor mice that received the patients’ fecal microbiota exhibited prolonged tremor time and impaired mobility. However, supplementation with *Lactobacillus plantarum* L5, the main GABA-producing bacterium, improved tremor and mobility, suggesting that GM plays an important role in essential tremor [51]. In addition, probiotics supplementation has been shown to reduce anxiety-like behaviors and autism spectrum disorder-like behaviors [52-54]. A meta-analysis found that individuals with IBDs are at a higher risk of developing AD compared to non-inflammatory bowel populations. Moreover, patients with a diagnosis of Crohn’s disease or ulcerative colitis are at a significantly higher risk of developing AD and PD [55]. Another study showed that the relative abundance of *Firmicutes* was significantly lower in patients with AD [56], while a reduction in *Lachnospiraceae* leads to a severe worsening of motor and non-motor symptoms in patients with PD [57, 58]. Furthermore, lower incidence of *Lactobacillus*, *Bifidobacteria*, and butyrate-producing bacteria in fecal microbiota were found in children with neurodevelopmental disorders [59]. In patients with cerebral autosomal dominant arteriopathy with subcortical infarcts and leukoencephalopathy (CADASIL), potential probiotics *Eubacterium eligens* and *Roseburia faecis*, were depleted, while *Fusobacterium varium* and *Clostridium aldenense* were enriched [60].

### 2.4 Gut Microbiota Closely Linked to Epilepsy

Recent studies have increasingly demonstrated the potential of GM in elucidating the pathogenesis of epilepsy and enhancing its treatment efficacy (Table **2**). The ketogenic diet (KD) has demonstrated efficacy in improving epilepsy, enhancing the presence of intestinal *Akkermansia muciniphila* and *Parabacteroides* in two mouse seizure models: *Kcna1^−/−^* mouse (for generalized tonic-clonic seizures) and the 6 Hz- induced mouse acute seizure model [61]. When *Kcna1*^−/−^ mice were transplanted with the GM from mice on KD, or specifically with *Akkermansia* and *Parabacteroides*, it was observed that both interventions attenuated epilepsy [61]. Interestingly, in GF mice following KD, seizure thresholds were not decreased when subjected to 6 Hz electrical stimulation. However, when *Akkermansia muciniphila* and *Parabacteroides* were transplanted to the gastrointestinal tracts of GF or antibiotic-treated mice alongside KD, the hippocampal GABA/glutamate ratio increased, and seizure thresholds significantly increased during electrical stimulation at 6 Hz. This suggests that the enriched gut flora associated with the KD possesses anti-epileptic properties [62]. Patients with idiopathic focal epilepsy exhibited notably elevated levels of *Campylobacter*, *Delftia*, *Haemophilus*, *Lautropia*, and *Neisseria* among *Proteobacteria*, alongside reduced levels of *Blautia*, *Coprococcus*, *Faecalibacterium*, and *Ruminococcus among Firmicutes*, compared to healthy volunteers [63]. Remarkably, in healthy volunteers, there was a significant increase in *Firmicutes*, which are primarily responsible for producing SCFAs, thereby potentially mitigating epilepsy risk. In children with focal epilepsy, *Actinobacteria, Escherichia/Shigella*, *Collinsella*, *Streptococcus*, and *Megamonas* were found to be enriched. Following a 3-month oxcarbazepine treatment, a significant reduction in these genera was observed [64]. Notably, *Escherichia/Shigella* and *Streptococcus* have been implicated in inducing the release of pro-inflammatory cytokines, including NLRP3 inflammasome, IL-6, and TNF-α. Additionally, *Collinsella* enrichment inhibits the growth of fermenting bacteria producing SCFAs and promotes the expression of IL-17, both contributing to epilepsy pathogenesis. Oxcarbazepine treatment may ameliorate epilepsy symptoms by reducing the abundance of these bacteria [64]. Furthermore, infants aged 1-4 years with refractory epilepsy exhibited significantly lower GM diversity, increased *Proteobacteria* and *Firmicutes,* and decreased *Bacteroidetes* compared to healthy counterparts [65]. Notably, *Proteobacteria* encompasses well-known pathogens such as *Escherichia*, *Salmonella,* and *Vibrio*, which may contribute to epilepsy pathogenesis. Following KD treatment, the level of *Proteobacteria* significantly decreased, and the abundance of *Bacteroidetes* approached that of healthy infants. In a follow-up study of 28 children with refractory epilepsy treated with KD, a reduction in the relative abundance of *Bifidobacteria longum* was observed. *Bifidobacteria longum* was positively correlated with the pro-inflammatory cytokine TNF, thus contributing to epilepsy [66].

## 3 THE MICROBIOTA-GUT-BRAIN AXIS AND EPILEPTOGENESIS

Epilepsy is a neurological disorder. A large number of studies have proved that GM can increase the susceptibility to epilepsy and even correlate with epileptogenesis (Table **3**). In a recent study, mice receiving microbiota from epileptic mice exhibited increased seizure susceptibility compared to those receiving microbiota from healthy mice. This study supports that alterations in GM affect the excitability of neurons in the brain, thereby leading to epilepsy through the gut-brain axis [67]. Results of a study exploring the effects of GM alterations on PTZ-induced mice seizure model demonstrated that intragastric administration of antibiotics exacerbated subsequent seizures. In contrast, intragastric administration of probiotics in this study attenuated subsequent seizures [68]. The relative abundance of *Firmicutes*, *Bacteroidetes*, *Lactobacillus* and *Ruminococcus* was significantly increased in the probiotics group, whereas it was significantly decreased in the antibiotics group. Their metabolites butyric acid, propionic acid and isovaleric acid were significantly increased in the probiotics group, whereas propionic acid and isovaleric acid were significantly decreased in the antibiotics group. Further studies suggest that this increased intestinal flora may reduce neuronal death in the brain and ameliorate subsequent PTZ-induced mice seizures by increasing butyric acid production and decreasing the expression of the death-related protein Bax in brain tissue. Another study investigated the relationship between intestinal inflammation and epilepsy in normal mice and dextran sulfate sodium-induced colitis mice. The findings suggest that alpha-lactoalbumin and sodium butyrate could exert anticonvulsant effects in PTZ-induced mice seizure models by decreasing intestinal inflammation, whereas intestinal inflammation reduced the antiepileptic effect of valproic acid [69]. Significant differences in the *Bacteroidetes/Firmicutes* ratio between WAG/Rij rats (a well-established genetic model of absence epilepsy) and Wistar rats have been found. Fecal microbiota transplantation (FMT) reduced seizure frequency in the WAG/Rij rats. This indicates that the gut-brain axis is an important pathway for epileptogenesis [70]. Results of a study comparing the GM composition and structure of rats with or without epilepsy before and after lateral fluid trauma (LFPI) showed that the risk of post-traumatic epilepsy (PTE) after craniocerebral trauma was moderately correlated with baseline fecal SCFA levels [71]. SCFAs, such as acetic acid, propionic acid, and butyric acid, are important metabolites produced by gut microbes, which protect against multiple aspects of epileptogenesis, including neurotransmitter modulation, oxidative stress and neuroinflammation, maintenance of BBB integrity, and attenuation of responses to psychosocial stress [72, 73].

Several observational clinical studies have shown that GM dysbiosis is considered a high-risk factor for neurological complications like epilepsy and seizures in some IBDs. These IBDs may contribute to epileptogenesis through the gut-brain axis pathway, such as immune activation, pro-inflammatory factors, and neurotransmitter release. In addition, GM dysbiosis can weaken the function of the intestinal immune barrier and disrupt the BBB, which in turn induces the production of inflammatory factors in the glial cells of the brain, leading to behavior and cognition impairments, lowering the seizure threshold or inducing epileptiform discharges, or even leading directly to seizures [74-77].

Taken together, these findings highlight that changes in the gut microbiome can lead to changes in their metabolites, intestinal barrier, and intestinal inflammation, all of which may contribute to epileptogenesis.

## 4 THE MICROBIOTA-GUT-BRAIN AXIS AND EPILEPSY DEVELOPMENT

Inflammation, metabolic changes, alterations in neuronal excitability, and gliosis induced by GM may play a crucial role in exacerbating epilepsy after its onset (Table **4**). According to a multi-omics analysis study, the GM profile of rats with lithium-pilocarpine-induced temporal lobe epilepsy significantly contributes to the maintenance of the epileptic state. This study showed increased pro-inflammatory *Desulfovibrio* and *Marvinbryantia,* along with increased excitatory neurotransmitters. Conversely, there was a notable reduction in the Lachnospiraceae family, which is involved in SCFA production. This reduction may lead to further neuroinflammation, and these inflammatory responses can worsen the progression of epilepsy [78]. In another study of lithium-pilocarpine-induced epilepsy rats, cannabidiol demonstrated the ability to attenuate GM dysbiosis and metabolic disorders in epileptic rats, potentially contributing to its anti-epileptic effects. Specifically, cannabidiol attenuated the reduction of butyric acid-producing bacteria such as *Prevotellaceae_UCG-001* and *Ruminococcaceae_UCG-005*, thereby exerting anti-inflammatory effects and improving epilepsy [79]. The U.S. Food and Drug Administration-approved vagus nerve stimulation for epilepsy treatment was found to improve GM, including the activation of intestinal glial cells, enhancement of intestinal barrier function, and stimulation of enteroendocrine cells to produce gut peptides and hormones. These substances are then absorbed into the bloodstream, affecting central events. This underscores the crucial role of GM in epilepsy development [32, 80].

A clinical study comparing the GM composition of drug-resistant epilepsy (DRE) patients, drug-sensitive epilepsy patients, and healthy controls revealed significant differences. The DRE patients exhibited distinct GM composition compared to the other two groups. Specifically, DRE patients showed a lower relative abundance of *Bacteroides finegoldii* and *Ruminococcus_g2*. *Bacteroides* species are able to produce the inhibitory neurotransmitter GABA associated with seizure control. *Ruminococcus_g2* may be involved in seizure control by modulating adenosine triphosphate binding cassette transporter (a transmembrane transporter protein pump that regulates the balance of excitability and inhibition in neurons) and N-acetyl aspartic acid, a marker of neuronal health. These GM differences could potentially serve as prognostic and efficacy assessment biomarkers during the development of epilepsy [81, 82]. Additionally, an analysis of different aspects of gastrointestinal function in epilepsy patients compared to healthy individuals indicated a higher incidence of functional gastrointestinal disorders among epilepsy patients. Furthermore, DRE patients had a greater likelihood of epileptic recurrence in the presence of altered bowel movements, especially insomnia [83]. In addition, some studies have shown that KD, which is commonly used in epilepsy treatment, may be used to control epilepsy by regulating GM [84]. These results suggest a clear correlation between the disruption of the normal composition of GM and the development of epilepsy.

## 5 THE MICROBIOTA-GUT-BRAIN AXIS AND EPILEPSY TREATMENT

**Fig. (2):**
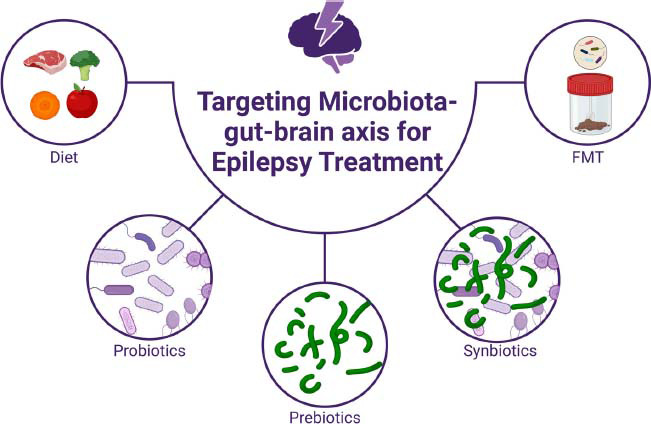
The microbiota-gut-brain axis and epilepsy treatment. Approaches that target the microbiota-gut-brain axis for the treatment of epilepsy include diet, probiotics, prebiotics, synbiotics, and FMT. Created with BioRender.com.

Emerging studies indicate that interventions aimed at modulating GM hold considerable promise in improving epilepsy treatment, particularly in cases of refractory epilepsy [85]. More importantly, compared to conventional ASMs, these methods may appear to have fewer side effects. With the in-depth research and understanding of the gut-brain axis, regulating gut microbes and their metabolites, including FMT, dietary modifications, probiotics, prebiotics, and synbiotics, will provide a new avenue for epilepsy treatment (Fig. **2**).

### 5.1 The Potential Application of Fecal Microbiota Transplantation in Epilepsy Treatment

FMT is an increasingly promising approach to rebuilding GM, demonstrating efficacy across various conditions, including epilepsy [62, 86, 87]. In 2017, He *et al.* first reported significant improvements in intestinal and neuronal symptoms after FMT in a female patient with Crohn's disease and epilepsy for 17 years. This opens a window for the application of the gut-brain axis concept to control epilepsy [88]. Further studies have revealed intriguing insights. For instance, transplanting fecal microbiota from chronically stressed rats into sham-stressed rats heightened susceptibility to epilepsy and prolonged seizure duration. Conversely, transplanting fecal microbiota from sham-stressed rats into chronically stressed rats counteracted the epileptic effects of stress. Remarkably, introducing fecal microbiota from non-stressed rats into chronically stressed rats reversed the seizure-promoting impacts of stress. This underscores the pivotal role of GM dysbiosis in stressed states, which exacerbates the kindling process within the basolateral amygdala. However, FMT aimed at rectifying this GM dysbiosis effectively countered its pro-epileptic effects [89].

### 5.2 Diet Modifications

Diet plays a crucial role in GM modulation, affecting brain structure and function through the gut-brain axis [90, 91]. The KD, a high-fat and low-carbohydrate diet, has shown efficacy in managing refractory epilepsy. Recent studies indicate that KD can modify GM composition, suggesting its therapeutic effect might be mediated through GM alterations [92-94]. For instance, a study involving Taconic Swiss Webster and Jackson C3HeB/FeJ *Kcna1^-/-^* mice revealed that KD reduced the alpha diversity of GMs and gamma-glutamyltranspeptidase activity while increasing the relative abundance of *Akkermansia muciniphila* and *Parabacteroides* [61]. These bacteria are associated with enhanced ketosis and metabolic improvements. The reduction in gamma-glutamyltranspeptidase activity leads to an increase in the GABA/glutamate ratio, offering protective effects against epilepsy. Notably, GF mice or those treated with antibiotics, lacking gut microbes, do not benefit from KD, underscoring the role of GM alterations in KD’s efficacy. In a study involving children with epilepsy on a 3-month KD, significant reductions in *Bifidobacteria* and *Eubacterium rectale* were observed [95]. However, based on current knowledge of the composition and function of GM, both bacteria and their metabolites, such as SCFAs, are considered beneficial to health. Their decreased abundance of KD may be attributed to the dietary restriction of carbohydrates, leading to ketone body production from fats as an alternative energy source. To counteract this reduction, supplementation with non-digestible carbohydrates like fibers may be beneficial for individuals with epilepsy on KD. Furthermore, research on high-fat diet-induced obesity models in mice has shown that polysaccharides from lycium barbarum, cultured cordyceps sinensis, and herbal medicines can modulate GM diversity and abundance while increasing the production of SCFAs, thereby inhibiting obesity [96-99]. Although no therapeutic studies have explored these polysaccharides’s efficacy in epilepsy, their potential to alter GM and metabolic pathways suggests a promising avenue for future research in epilepsy treatment.

### 5.3 The Application of Probiotics, Prebiotics, and Synbiotics in the Treatment of Epilepsy

Probiotics are live microorganisms beneficial to the host and are considered a promising one-size-fits-all strategy to modulate GM due to their low toxicity [100-102]. They produce beneficial metabolites such as SCFAs, serotonin, and neurotransmitters, which exert neuroprotective effects through the gut-brain axis, making them a promising therapeutic approach for gastrointestinal and brain dysfunction [103]. For instance, oral administration of *Bifidobacterium longum* to rats with lithium-pilocarpine-induced temporal lobe epilepsy rats for 30 days resulted in reduced neuronal death in the amygdala and anxiety levels in rats. Studies have shown that *Bifidobacterium longum* increased the expression of anti-inflammatory and neuroprotective genes, suggesting the potential of this bacterium for use in the treatment of epilepsy [104]. Bagheri *et al*. reported that in the PTZ-induced rat epilepsy model, the mixed probiotics of *Lactobacillus rhamnosus*, *Lactobacillus reuteri,* and *Bifidobacterium infantis* could significantly reduce the severity of seizures and improve spatial learning and memory ability by regulating GABA concentration, nitric oxide, malondialdehyde, and total antioxidant capacity [105]. Additionally, patients with temporal lobe epilepsy taking BIFICO probiotic capsules consisting of *Bifidobacterium longum, Lactobacillus acidophilus*, and *Enterococcus faecalis* exhibited reduced seizures, as well as significant improvements in anxiety, depression, and low quality of life indicators. This may be due to the fact that these probiotics affect temporal lobe epilepsy pathology by modulating metabolism and neurotransmitters, including dopamine, serotonin, norepinephrine, and GABA, through the gut-brain axis [106].

Prebiotics are indigestible food components that stimulate the growth and activity of probiotics [107-109]. Probiotics and prebiotics can also be used in combination as synbiotics. In a study, treatment with *Bifidobacterium bifidum* and *Lactobacillus lactis* as probiotics, inulin as a prebiotic, and their combination as a symbiotic improved spontaneous seizures and cognitive impairment in kainic acid-induced status epileptic rats. Synbiotics seem to work better than probiotics or prebiotics alone [110]. These interventions also inhibited oxidative stress and inflammatory responses in the hippocampus, correlating with their therapeutic effects [111].

In summary, probiotics, prebiotics, and synbiotics offer promising avenues for epilepsy treatment by modulating the gut-brain axis, such as neurotransmitters and inflammatory responses. Further research in this area holds significant potential for developing novel therapeutic strategies for epilepsy management.

## CONCLUSIONS AND FUTURE PERSPECTIVES

The establishment of the gut-brain axis begins with the colonization of the GM at birth, closely associated with neurological development and brain physiology. In epilepsy research, GM dysbiosis has emerged as a significant characteristic, potentially informing epilepsy prevention and diagnosis. Moreover, both the modulation and intervention of GM have shown antiepileptic effects, which may provide a new avenue to develop AEDs. However, the causal relationship between the altered GM and epileptogenesis remains a topic of debate. Before implementing GM modulation as a target for AEDs in epilepsy treatment, researchers need to address various challenges related to the complexity of the gastrointestinal tract and individual health.

Several critical aspects need to be addressed to establish and elucidate this causal relationship, as well as to improve the feasibility of designing AEDs targeting the gut-brain axis. Firstly, it is crucial to characterize the GM during the latency period and periods of epileptogenesis and epilepsy development. This approach helps to clarify whether the specific GM alterations lead to epileptogenesis and epilepsy development. However, most of the current studies have focused on clarifying the relationship between GM alterations and epilepsy susceptibility, as well as GM alterations between epileptic and normal individuals. Studies related to the differences in GM from the latency period to the developmental process are not abundant. Therefore, a substantial amount of research is needed in this area. Secondly, considering that GM is susceptible to a variety of conditions, future studies of gut-brain axis mechanisms in epileptogenesis and epilepsy development should consider GM differences in different types of epilepsy. Understanding the common GM alterations in different types of epilepsy will greatly help to reveal the specific mechanism of GM on epilepsy. Thirdly, in order to reduce the influence of other factors associated with altered GM, there is a need to use more rigorous experimental designs on the one hand and to validate the effects of specific gut microbes and their metabolites on epilepsy on the other hand, which can also contribute to epilepsy treatment. Butyrate, a major intestinal metabolite, has been highlighted in numerous studies for its role in epilepsy pathogenesis and treatment. Notably, butyrate has been shown to upregulate melatonergic pathways in intestinal epithelial cells [112]. Intriguingly, this upregulation extends to brain cells, particularly in astrocytes, which are known to efflux melatonin [113]. Such local melatonin production has the capacity to suppress inflammatory activity and oxidative stress to optimize mitochondrial function [114]. These are all crucial aspects of epileptogenesis [115]. Recent investigations further underscore the interplay between gut-derived butyrate and local melatonin in the pathophysiology of AD [116], which is a condition intricately linked to seizure emergence [117]. Additionally, sodium butyrate, as a pan-histone deacetylase inhibitor, exerts anti-inflammatory effects by inhibiting NLRP3 inflammasome activation [118]. Furthermore, butyrate counteracts the inhibition of astrocytic excitatory amino acid transporters (EAAT) 1 and EAAT2 by Yin Yang (YY)1 and its corepressors histone deacetylase [119]. These can provide deeper insights into the role of the gut-brain axis in epilepsy and facilitate the development of targeted therapeutic interventions.

Taken together, defining specific GM alterations can be challenging due to variations in experimental replication. However, efforts should be made to ensure consistent experimental conditions. Fortunately, the advancement of high-throughput sequencing and analysis platforms enables the verification of results from multiple perspectives, leading to more accurate outcomes. Microbiomics combined with metabolomics, transcriptomics, proteomics, and single-cell RNA sequencing provide insights into the mechanisms of the gut-brain axis and epilepsy. As our understanding of the bidirectional communication between the brain and the gut improves, it will provide an entirely new avenue for the development of new anti-epileptic drugs.

## Figures and Tables

**Table 1:** The microbiota-gut-brain axis and neurological disorders.

**References**	**Experimental Subject**	**Result**
Lin *et al*. [48]	PTZ-induced mice seizure model	*Klebsiella pneumoniae increased* susceptibility to epilepsy
Dong *et al*. [49]	Epilepsy patients	↑ *Fusobacterium*, *Verrucomicrobia*, *Alloprevotella*
Chen *et al*. [50]	GF 3×Tg mice and SPF 3×Tg mice	GM is essential for AD pathological changes
Zhong *et al*. [51]	Tremor patients and mice	*Lactobacillus plantarum* L5 improved tremor and mobility
Chen *et al*. [56]	AD patients	↓ *Firmicutes*
Ma *et al*. [57, 58]	PD patients	↓ *Lachnospiraceae*
Bojović *et al*. [59]	Children with neurodevelopmental disorders	↓ *Lactobacillus*, *Bifidobacteria*, butyrate-producing bacteria
Liu *et al*. [60]	CADASIL patients	↑ *Fusobacterium varium*, *Clostridium aldenense*; ↓ *Eubacterium eligens*, *Roseburia faecis*

**Note**: ↓: Decreased, ↑: Increased.

**Table 2:** Gut microbiota alterations in epilepsy.

**References**	**Types of Epilepsy**	**Result**
Olson *et al*. [61]	*Kcna1*^−/−^ mice model for spontaneous tonic clonic seizures treated with ketogenic diet	↑ *Akkermansia Muciniphila, Parabacteroides*
Olson *et al*. [61]	6 Hz-induced mouse acute seizure model treated with ketogenic diet	↑ *Akkermansia Muciniphila, Parabacteroides*
Şafak *et al*. [63]	Patients with idiopathic focal epilepsy	↑ *Campylobacter, Delftia, Haemophilus, Lautropia, Neisseria*; ↓ *Blautia*, *Coprococcus*, *Faecalibacterium*, Ruminococcus
Zhou *et al.* [64]	Children with focal epilepsy	↑ *Actinobacteria*, *Escherichia/Shigella*, *Collinsella*, *Streptococcus*, *Megamonas*
Zhou *et al.* [64]	Children with focal epilepsy treated with oxcarbazepine	↓ *Actinobacteria*, *Escherichia/Shigella*, *Collinsella*, *Streptococcus*, *Megamonas*
Xie *et al.* [65]	Infants aged 1-4 years with refractory epilepsy	↑ *Proteobacteria*, *Firmicutes*
Xie *et al.* [65]	Infants aged 1-4 years treated with refractory epilepsy	↑ *Bacteroidetes*; ↓ *Proteobacteria*
Dahlin *et al.* [66]	Children with drug-resistant epilepsy treated with ketogenic diet	↓ *Bifidobacteria longum*, *Bifidobacteria breve*

**Note**: ↓: Decreased, ↑: Increased.

**Table 3:** The microbiota-gut-brain axis and epileptogenesis.

**References**	**Experimental Subject**	**Result**
Mengoni *et al*. [67]	GM transplanted from epileptic animals to healthy recipient animals	Recipient animals were more susceptible to seizures
Zhai *et al*. [68]	PTZ-induced mice seizure model	Antibiotics exacerbated seizures; Probiotics improved seizures
De Caro *et al*. [69]	PTZ-induced epileptic mice	Intestinal inflammation increases susceptibility to epilepsy
Citraro *et al*. [70]	WAG/Rij rats	FMT reduced seizure frequency in WAG/Rij rats
Medel-Matus *et al*. [71]	Rats with LFPI	Susceptibility to epilepsy was associated with GM characteristics before LFPI

**Table 4:** The microbiota-gut-brain axis and epilepsy development.

**References**	**Experimental Subject**	**Result**
Oliveira *et al*. [78]	Lithium-pilocarpine induced rat temporal lobe epilepsy	GM profile of epileptic rats contributes to the maintenance of the epilepsy state. Temporal lobe epilepsy in rats: ↑ *Desulfovibrio*, *Marvinbryantia*, L-glutamic acid levels; ↓ Lachnospiraceae family
Gong *et al*. [79]	Lithium-pilocarpine induced rat temporal lobe epilepsy	Cannabidiol improves epilepsy by increasing *Prevotellaceae_UCG-001* and *Ruminococcaceae_UCG-005*
Peng *et al*. and Lee *et al*. [81, 82]	Epilepsy patients	DRE patients: ↓ *Bacteroides finegoldii*, *Ruminococcus_g2*
Avorio *et al*. [83]	Epilepsy patients	Epilepsy patients have a higher incidence of functional gastrointestinal disorders; patients with DRE have an increased rate of seizure recurrence when bowel movements alter.

## LIST OF ABBREVIATIONS

AD: Alzheimer's Disease
AEDs: Antiepileptic Drugs
ANS: Autonomic Nervous System
ASMs: Antiseizure Medications
BBB: Blood-brain Barrier
CADASIL: Cerebral Autosomal Dominant Arteriopathy with Subcortical Infarcts and Leukoencephalopathy
CNS: Central Nervous System
DRE: Drug-resistant Epilepsy
ENS: Enteric Nervous System
FMT: Fecal Microbiota Transplantation
GABA: Gamma-aminobutyric Acid
GF: Germ-free
GM: Gut Microbiota
Gut-brain Axis: Microbiota-gut-brain Axis
HPA: Hypothalamic-pituitary-adrenal
5-HT: 5-Hydroxytryptamine
IBDs: Inflammatory Bowel Diseases
IL: Interleukins
KD: Ketogenic Diet
LFPI: Lateral Fluid Percussion Injury
LPS: Lipopolysaccharide
PD: Parkinson’s Disease
PTE: Post-traumatic Epilepsy
PTZ: Pentylenetetrazole
SCFAs: Short-chain Fatty Acids
SD: Sprague Dawley
SPF: Specific Pathogen Free
TNF: Tumor Necrosis Factor
VN: Vagus Nerve
